# Premenstrual dysphoric disorder and exacerbation of affective and psychotic disorders: prevalence and diagnostic challenges

**DOI:** 10.1192/j.eurpsy.2026.10329

**Published:** 2026-07-31

**Authors:** T. Reilly

**Affiliations:** Department of Psychiatry, University of Oxford, Oxford, United Kingdom

## Abstract

**Abstract:**

Premenstrual dysphoric disorder, included for the first time in DSM-5 and ICD-11, is defined by the timing of affective symptoms in relation to the menstrual cycle. Gold-standard diagnosis involves prospective charting of symptoms, which must be confined to the premenstrual phase and minimal/absent at other times. Using this diagnostic method, the pooled prevalence of premenstrual dysphoric disorder is 3.2%, a much smaller proportion than experience broadly define premenstrual syndromes. Correct diagnosis is important as evidence-based treatments, including both selective serotonin reuptake inhibitors and hormonal therapies, are available. Additionally, a range of mental and physical disorders demonstrate menstrual exacerbation, deterioration of symptoms linked with the menstrual cycle. Depression, bipolar disorder and schizophrenia all have evidence of menstrual worsening. I will discuss diagnosis and treatment implications for these menstrual-related disorders.

**Disclosure of Interest:**

None Declared

